# Effects of Athletic Trainer Direct Employment on the Management of Sports-Related Injuries in High School Athletes

**DOI:** 10.7759/cureus.32995

**Published:** 2022-12-27

**Authors:** Rock P Vomer, Emma York, Michael T Kalkbrenner, Zachary Kershaw, LaRae L Seemann, George G. A Pujalte

**Affiliations:** 1 Departent of Family and Community Health, Department of Orthopedics, Division of Sports Medicine, Duke University, Durham, USA; 2 Family Medicine, Eastern Virginia Medical School, Norfolk, USA; 3 Counseling and Educational Psychology, New Mexico State University, Las Cruces, USA; 4 Athletic Training, Phillips Academy, Andover, USA; 5 Family Medicine, Mayo Clinic Jacksonville Campus, Jacksonville, USA; 6 Departments of Family Medicine, and Orthopedics and Sports Medicine, Mayo Clinic, Jacksonville, USA

**Keywords:** high school sports, scope of practice, practice management, school health promotion, athletic injuries

## Abstract

Background: Hiring athletic trainers (ATs) in high schools has attracted rising interest as a potential way of improving adolescents' health by enhancing their safety and reducing their risk of injury.

Objective: This study aims to determine if there is a difference in the referral patterns, injury diagnoses, and injury treatments performed at a metropolitan high school when an AT is employed versus not employed by the school.

Design: This is a retrospective quantitative two-period study.

Setting: The study was conducted in the high school athletic department in Norfolk, Virginia, and the study population was high school athletes (age 14-18).

Main outcome measures: Changes in referral patterns, injury diagnoses, and injury treatments performed at a local high school when an AT is employed versus not employed by the school; specifically, we examined the number of and percent changes in yearly treatments, referrals, evaluations, and re-evaluations during the two periods.

Results: Our first t-test revealed a statistically significant increase in the number of reported injuries between 2011-2015 (M = 58.00, SD = 44.86) and 2016-2020 (M = 299.00, SD = 40.93, p = 0.006. The second t-test revealed a statistically significant increase in the number of referrals between 2011-2015 (M = 249.00, SD = 353.41) and 2016-2020 (M = 1188.00, SD = 158.21), p = 0.014. The third t-test revealed a statistically significant increase in the number of treatment items between 2011-2015 (M = 150.67, SD = 175.32) and 2016-2020 (M = 636.67, SD = 211.72), p = 0.01.

Conclusions: The present study found an increased frequency of reported injuries, referrals, and treatment after ATs directly joined the staff of a large metropolitan high school. These findings suggest that direct employment of ATs is associated with greater recording of injuries and treatment of conditions. A reduction in referrals occurs with the presence of directly employed ATs, which could result in improved health for student-athletes, but this needs further study.

## Introduction

Athletic trainers (ATs) are healthcare professionals who work in collaboration with physicians to provide a range of services, including injury and illness prevention, evaluation, diagnosis, treatment, and rehabilitation, as well as emergency care, to physically active individuals [1]. Hiring ATs in high schools has attracted rising interest as an approach to improving adolescents' health by enhancing their safety and reducing their risk of injury [1,2]. Several recent studies suggest that high schools with an AT on staff report greater recognition of concussions and lower rates of musculoskeletal injury and re-injury [3]. These schools are also more likely than comparators to have athletics-specific emergency action plans [4,5].

Several associations, including the American Medical Association, the American Academy of Pediatrics Council on Sports Medicine and Fitness, the National Federation of State High School Associations, and the Appropriate Medical Care for Secondary School-Aged Athletes Task Force, have recommended that an AT be available to provide medical care for secondary school-aged athletes [5-7]. Despite these recommendations, only 66% of US high schools provide any form of AT services to student-athletes [7,8]. A recent report found that of the schools with AT services, 53% received full-time services, while 47% received part-time services, and of these schools, 57% of the ATs were employed by external medical or university employers. Barriers to providing AT services include, but are not limited to, budgetary constraints, school size, and lack of awareness of the role of an AT. For this reason, many schools do not utilize ATs, though evidence suggests direct employment saves Medicaid tax dollars [8-10].

External ATs have limited access to student-athletes during the day compared to directly employed ATs, who have access to student-athletes all day. Furthermore, the availability of only one AT at a high school frequently means that an AT is unable to be physically present when multiple sporting events overlap. Such staffing shortages limit the AT's ability to provide basic but invaluable training and education to the coaching staff regarding basic emergency care [11]. A recent study demonstrated that Medicaid costs are $64 dollars higher per person in schools that do not have access to ATs [6]. Additionally, these schools are less likely to have emergency action plans [2].

Because ATs possess a specific skill set and work in close collaboration with physicians, they play a vital role in coordinating and expediting care as deemed medically appropriate [12]. In addition, ATs who work in the high school setting are uniquely positioned to evaluate, treat, and rehabilitate injuries [9]. Being timely and present creates an opportunity for ATs to help reduce both the risks and effects of injuries and associated medical costs [13-15].

The purpose of this study was to determine if there is a difference in the referral patterns, injury diagnoses, and injury treatments performed at a local high school when an AT is employed versus not employed by the school. We hypothesize that in direct employment.

## Materials and methods

Study design 

We conducted a retrospective review of de-identified archival data from the SportsWare™ EMR (Medco Sports Medicine, Tonawanda) electronic medical record used by the High School Athletics department, part of the Norfolk (VA) Public School system. No personally identifying data of any type was collected at any time. The procedure of the study was as follows: the total number of de-identified records and the aggregate number of evaluations, injuries, treatments, and referrals that occurred between 2011 and 2020 were collected from SportsWare. The pre-period for this study was 2011-2015, when the school district did not directly employ ATs, and the post-period was 2016-2020, when ATs were directly employed. A total of 2,177 athletes participated in the pre-period (i.e., before the ATs were hired), and 2,109 athletes participated in the post-period (i.e., after the ATs were hired). The same sports were offered at the high school in the pre and post-periods. Participants’ demographic data for gender identity, ethnic identity, and age were not available. Data were compared between these two periods to determine if there were changes in practice patterns.

Data collection 

The data points we collected were the total number of injuries, the total number of initial evaluations by ATs, the total number of re-evaluations, the total number of treatment items, and the total number of referrals. The inclusion criteria for this study included high school athletes, aged 14-18, and evaluation, treatment, re-evaluation, or referrals by ATs during the study period. For this study, there was only one athletic trainer entering data per time period. We excluded data prior to 2011 and injuries that happened outside organized high school sports. We additionally excluded any instances of incomplete medical record documentation of an injury, treatment, or referral.

Data analysis

The present study included one categorical level independent variable (time) with two levels: pre-period (2011-2015) and post-period (2016-2020). There were three dependent variables measured on continuous-level scales, including the number of reported injuries, the number of referrals, and the number of treatment items. The frequency of injury was defined as the number of injuries per year. The number of referrals was defined as any referral to an outside healthcare provider. Treatment items were defined as any rehabilitation service provided by the AT inside the high school. Statistical tests of mean comparisons for one categorical level independent variable with two levels (t-test) were performed using IBM SPSS version 26 with alpha set at .05 (IBM Corp., Armonk, NY) [15]. The Eastern Virginia Medical School Institutional Review Board determined that this project was exempt from review.

Three paired samples t-tests were computed to investigate mean differences in the frequency of injuries, referrals, and treatment items between the pre-test period (2011-2015) and the post-test period (2016-2020). The analyses were run separately because the aim of the study was to examine differences over time across three independent outcome variables: frequency of injuries, number of referrals, and number of treatment items. In addition, our data represents thousands of participants; however, the archival nature of the data only allowed us to gather data points for each calendar year. Thus, our sample size (actually recorded data points) was not large enough to compute a single repeated-measures multivariate analysis. A Bonferroni correction was applied to control for the familywise error rate. Based on the recommendations of the field (2018), Cohen’s d was computed to estimate the size of the effect of the t-test results. Cohen’s d is computed by taking the difference between the mean score of each group divided by the pooled standard deviation. We referred to the following guidelines provided by Sink and Mvududu to interpret Cohen’s d: d < 0.20 = small, d = 0.30 to d = 0.7 = medium, and d < 0.80 large [16].

## Results

Three t-tests were computed to investigate differences in the frequency of injuries, referrals, and treatment items between the pre-test period (2011-2015) and the post-test period (2016-2020). Consistent with the protocol for reporting t-test results [15], we report the overall mean and standard deviation scores for the pre- and post-AT hiring periods (Table 1).

**Table 1:** T-test results comparing reported injuries, number of referrals, and treatment items between the pre- and post-periods

Outcome variable	Estimate	Pre-period (2011–2015)	Post-period (2016–2020)
Reported injuries	Mean	58	299
	Standard deviation	44.86	40.93
	p-value	0.006	0.006
	Cohen's d	5.61	5.61
Referrals	Mean	249	1188
	Standard deviation	353.41	158.21
	p-value	0.014	0.14
	Cohen's d	3.43	3.43
Treatment items	Mean	150.67	636.67
	Standard deviation	175.32	211.72
	p-value	0.011	0.011
	Cohen's d	2.5	2.5

The first t-test was computed to investigate differences in the frequency of injuries between 2011-2015 and 2016-2020. Results revealed a statistically significant increase in the number of reported injuries between 2011-2015 (M = 58.00, SD = 44.86) and 2016-2020 (M = 299.00, SD = 40.93), 95% CI [128.58, 353.42], t(3) = 6.82, p = 0.006, d = 5.61. During the pre-period (2011-2015), 13% (n = 295) reported an injury. During the post-period (2016-2020) 57% (n = 1,196) reported an injury. A second t-test was run to determine if there were differences in referrals between 2011-2015 and 2016-2020. Results revealed a statistically significant increase in the number of referrals between 2011-2015 (M = 249.00, SD = 353.41) and 2016-2020 (M = 1188.00, SD = 158.21), 95% CI [309.82, 1568.18], t(4) = 4.14, p = 0.014, d = 3.43. A final t-test was run to determine if there were differences in treatment items between 2011-2015 and 2016-2020. Results revealed a statistically significant increase in the number of treatment items (e.g., evaluations, re-evaluations, rehabilitation exercises, splinting, taping, and pain modalities) between 2011-2015 (M = 150.67, SD = 175.32) and 2016-2020 (M = 636.67, SD = 211.72), 95% CI [260.94, 711.06], t(2) = 9.29, p = 0.011, d = 2.50.

## Discussion

The findings of the present study demonstrated statistically significant increases in the frequency of reported injuries, referrals, and treatment items between the pre-test period (2011-2015) and the post-test period (2016-2020). The effect sizes (practical significance) of the results were all in the very strong range based on previously published guidelines [15]. This suggests that in direct employment models, athletic trainers may be better equipped to identify, document, treat, and manage injuries that occur in high school athletes compared to externally employed athletic trainers.

The methodological limitations of the present study should be considered when interpreting the findings. We collected data from one high school located in the Norfolk County area; this comes with the caveat that our findings might not generalize to high schools in other locations. To address this, a larger study with more schools could be conducted. Moreover, the archival nature of our data collection method limited access only to demographic variables. Accordingly, we were not able to examine potential interaction effects between demographic variables and ATs across outcome variables (injury rates, treatment items, and referrals). Future researchers can extend this line of inquiry by recruiting a larger sample and testing for multivariate interaction effects by demographic variables, for example, age, gender identity, ethnic identity, and sport.

ATs provide a variety of services to the athletes they work with, including diagnosis, treatment, and evaluation of injuries. In support of this, numerous professional societies, including the American Medical Association, the American Academy of Pediatrics Council on Sports Medicine and Fitness, the National Federation of State High School Associations, and the Appropriate Medical Care for Secondary School-Aged Athletes Task Force, have recommended that an AT be available to provide medical care for secondary school-aged athletes [5-7]. Despite these recommendations, US high schools are not required to have an AT on staff regardless of the number of athletes at the school, and only 66% of US high schools provide any form of AT services to student-athletes. High schools that lack adequate AT services are more likely to see the following in their athletes: increased musculoskeletal injuries, lower rates of concussion recognition, lower rates of emergency action plans, and a higher cost of care for patients covered by Medicaid. Of the high schools that do have access to AT service, 57% are employed through external universities or medical groups, and based on a report by Barter et al., 45.8%±24.3 these ATs are employed part-time [17]. The wide range of availability may contribute to missed identification of injuries, prevention, and treatment opportunities. In situations where a high school does not have adequate access to AT services, athletes must go off-site for treatments that could have been rendered on-site, likely increasing the cost of care. 

## Conclusions

Our study sought to determine if there is a difference in the referral patterns, injury diagnoses, and injury treatments performed at a local high school when an AT is employed versus not employed by the school. We found increases in the frequency of reported injuries, referrals, and treatment items. Although this study is limited to data from only one high school, the difference between the pre- and post-test groups was very large. Collectively, the results of this exploratory study suggest that the direct employment of ATs might have utility in promoting safety for high school athletes. Future research is necessary to continue to examine the work model of AT employment at high schools and other venues to quantify its value and identify areas for improved deployment of ATs.
